# Supplementary material for: Complexome profiling of the Chlamydomonas psb28 mutant reveals TEF5 as an early PSII assembly factor
Source: Plant Cell. 2025 Mar 17;37(6):koaf055. doi: 10.1093/plcell/koaf055 (PMC12164590; doi:10.1093/plcell/koaf055)
Supplement: koaf055_Supplementary_Data [file koaf055_supplementary_data.zip › psb28_interactive.zip!/plots/intensity/qualityPlots/peptides/NUO8_Cre12.g496750.t1.2.html]

### Cre12.g496750.t1.2

Trivial name: NUO8  
SeqCoverage [median]: 32.50  
Mass: 26.50 kDa  
  
 

| Sequence | MissedCleavage | Mass [Da] | wtIntensity | muIntensity | Score |
| --- | --- | --- | --- | --- | --- |
| EELLYDK | 0 | 908.45 | 7957.63 | 1142.42 | 104.45 |
| EELLYDKQK | 1 | 1164.60 | 0.00 | 3188.29 | 44.20 |
| GMAYTLGAFFDKK | 1 | 1447.72 | 10114.97 | 3742.87 | 80.31 |
| LLENGDKWETEIATNLR | 1 | 2001.02 | 891.28 | 0.00 | 52.89 |
| RYPTGEER | 1 | 1006.48 | 395.50 | 0.00 | 74.30 |
| TESLYR | 0 | 767.38 | 17121.72 | 14681.48 | 149.11 |
| VTIMYPFEK | 0 | 1126.57 | 16889.10 | 5464.03 | 146.79 |
| YASDWENDPTFK | 0 | 1471.63 | 800.29 | 0.00 | 62.30 |
| YASDWENDPTFKR | 1 | 1627.73 | 8599.53 | 8281.58 | 107.53 |
| YPTGEER | 0 | 850.38 | 1749.14 | 0.00 | 94.66 |
|  |  |  | 64519.2 | 36500.7 |  |
